# TDP-43 Is Not a Common Cause of Sporadic Amyotrophic Lateral Sclerosis

**DOI:** 10.1371/journal.pone.0002450

**Published:** 2008-06-11

**Authors:** Rita J. Guerreiro, Jennifer C. Schymick, Cynthia Crews, Andrew Singleton, John Hardy, Bryan J. Traynor

**Affiliations:** 1 Laboratory of Neurogenetics, National Institute of Aging, National Institutes of Health, Bethesda, Maryland, United States of America; 2 Center for Neuroscience and Cell Biology, University of Coimbra, Coimbra, Portugal; 3 Department of Physiology, Anatomy and Genetics, University of Oxford, Oxford, United Kingdom; 4 Reta Lila Weston Institute and Department of Molecular Neuroscience, Institute of Neurology, London, United Kingdom; 5 Reta Lila Weston Institute and Department of Neurodegenerative Disease, Institute of Neurology, London, United Kingdom; 6 Neurogenetics Branch, National Institute of Neurological Disorders and Stroke, National Institutes of Health, Bethesda, Maryland, United States of America; National Institutes of Health, United States of America

## Abstract

**Background:**

TAR DNA binding protein, encoded by *TARDBP*, was shown to be a central component of ubiquitin-positive, tau-negative inclusions in frontotemporal lobar degeneration (FTLD-U) and amyotrophic lateral sclerosis (ALS). Recently, mutations in *TARDBP* have been linked to familial and sporadic ALS.

**Methodology/Principal Findings:**

To further examine the frequency of mutations in *TARDBP* in sporadic ALS, 279 ALS cases and 806 neurologically normal control individuals of European descent were screened for sequence variants, copy number variants, genetic and haplotype association with disease. An additional 173 African samples from the Human Gene Diversity Panel were sequenced as this population had the highest likelihood of finding changes. No mutations were found in the ALS cases. Several genetic variants were identified in controls, which were considered as non-pathogenic changes. Furthermore, pathogenic structural variants were not observed in the cases and there was no genetic or haplotype association with disease status across the *TARDBP* locus.

**Conclusions:**

Our data indicate that genetic variation in *TARDBP* is not a common cause of sporadic ALS in North American.

## Introduction

Amyotrophic lateral sclerosis (ALS, OMIM #105400) is a rare and devastating neurodegenerative disorder of unknown etiology characterized by rapidly progressive paralysis leading to death due to respiratory failure, typically within 3–5 years of symptom onset. Population-based epidemiological studies of the disease show that between 1.6% and 5.7% of cases are familial in nature, whereas the remaining 95% occur sporadically throughout the population [1],[2]. Various genes that cause familial ALS have been identified, including copper/zinc superoxide dismutase [3], dynactin 1 [4] [5], alsin [6], senataxin [7], and vesicle-associated protein B [8]. In contrast to familial ALS, the genetics of the sporadic form are poorly understood and it is not known if sporadic ALS is monogenic (i.e., a single-gene disorder), polygenic (i.e., multiple interacting genes), multi-factorial (i.e., interacting genetic and environmental factors), or arises from some unknown non-genetic cause [9].

The neuropathology of ALS is characterized by the abnormal accumulation of insoluble ubiquitin proteins in the cytoplasm of degenerating motor neurons [10]. Recently, TAR DNA-binding protein 43 (*TARDBP*, OMIM #605078) was recognized as a major constituent of these neuronal cytoplasmic inclusions [11],[12],[13]. TDP-43 protein is evolutionary conserved and its structure consists of a glycine-rich domain and two RNA recognition motifs [14]. The exact function of TDP-43 remains unclear, though it is known to bind DNA and RNA (such as human immunodeficiency virus type 1 TAR DNA sequence motifs) [15], and to be involved in the regulation of messenger RNA splicing and exon skipping [16]. Four studies have independently reported *TARDBP* mutations in familial forms of ALS displaying autosomal dominant inheritance and in sporadic ALS. An A315T mutation in exon 6 of *TARDBP* was reported to segregate with disease within a multi-generational ALS family and this sequence variant was not found in 1,505 control subjects [17]. In other study, a missense mutation M337V in exon 6 of *TARDBP* segregated with disease within an ALS family across two generations. In addition, two missense mutations (Q331K and G294A) were identified in two of 372 sporadic ALS cases screened for mutations (representing 0.5% of the total cohort). None of the three mutations were identified in 1,262 control subjects [18]. In a separate study of 200 individuals with ALS from France and Quebec, eight additional sequence variants were identified in *TARDBP* including the previously identified A315T missense mutation and A382T in small families and D169G, G287S, G348C, R361S, N390D and N390S in individual sporadic cases [19]. In the latest study, two additional missense mutations (G290A and G298S) were identified in familial ALS cases [20]. None of the sequence variants identified in sporadic ALS patients have been seen in more than one case.

In this study, we undertook mutational screening of *TARDBP* in a cohort of 279 North American sporadic ALS cases and in a cohort of 806 ethnically-matched controls. The same case-control cohort had been previously genotyped using Illumina HumanHap550 BeadChips which assay over 550,000 tagged SNPs across the human genome [9]. This genotype data was analyzed to identify (a) genomic structural variants of the *TARDBP* locus relevant to the pathogenesis of sporadic ALS, (b) genetic association of SNPs within *TARDBP* with disease status, and (c) common haplotypes across the *TARDBP* locus that alter risk of motor neuron degeneration. Finally, we sequenced *TARDDP* in the African samples of the Human Gene Diversity Panel (n = 173) to examine genetic diversity of this gene in non-Caucasian populations.

## Materials and Methods

### ALS and control series

The case cohort used in this study consisted of 279 white, non-Hispanic subjects diagnosed with probable or definite sporadic ALS [21]. Of these, 181 were men and 98 were women and the average age of symptom onset was 55 years (range, 19–82 years). 58 patients described bulbar-onset disease, 217 patients presented with spinal-onset disease, 2 patients had generalized symptoms at onset and the remaining 2 patients presented with respiratory symptoms. These samples are publicly available from the NINDS Neurogenetics Repository at the Coriell Institute for Medical Research, NJ, USA (www.coriell.org).

The control cohort comprised of 806 neurologically normal white, non-Hispanic individuals obtained from the same NINDS Neurogenetics repository. These samples are available as precompiled panels (NDPT002, NDPT006, NDPT009, NDPT019 to NDPT024). None of the control samples had a medical history of ALS, Alzheimer's disease, ataxia, autism, bipolar disorder, brain aneurysm, dementia, dystonia, or Parkinson's disease and none had any first-degree relative with a known primary neurological disorder. The control and case cohorts were drawn from the same ethnic origin (i.e. Caucasian) at different clinical sites throughout the United States, but the control samples were not matched by age or sex with the ALS samples [9]. The control cohort consisted of 335 men and 471 women and the mean age at sample collection was 59 years (range 15–95 years).

An additional series of 173 anonymous African samples that are part of the Human Gene Diversity Panel (HGDP) [22] were included in the mutational analysis as controls to evaluate the genetic variability of *TARDBP* in non-Caucasian populations. These samples originated from eight different African populations, namely Biaka Pygmy (n = 35), Mozabite (n = 26), Bedouin (n = 25), Mandenka (n = 24), Yoruba (n = 23), Bantu (n = 20), Mbuti Pygmy (n = 13) and San (n = 7). All patients and controls gave written informed consent to participate in the study.

### DNA Sequencing

All the coding exons and 30bp of the flanking intron-exon boundaries of *TARDBP* (NM007375.3) were PCR amplified using primers designed using Primer3 software (available upon request) and Roche FastStart PCR MasterMix polymerase (Roche Diagnostics Corp., IN). Each PCR product was sequenced using Applied Biosystems BigDye terminator v3.1 sequencing chemistry and run on an ABI3730xl (Applied Biosystems, CA) genetic analyzer as per manufacturer's instructions. The sequences were analyzed with Sequencher software, version 4.2 (Genecodes, VA).

### SNP chip genotyping

All 279 US ALS samples included in this study had been previously genotyped with Illumina Infinium II HumanHap550 SNP chips (Illumina, San Diego, CA, USA) as part of a whole genome association study of sporadic ALS [9]. DNA from 594 control subjects had also been genotyped using HumanHap550 SNP chips. The remaining 212 control individuals were assayed with both Illumina Infinium II HumanHap240S SNP chips [9] and HumanHap317 SNP chip [23] and the data from both these chips were combined to provide a final control genotyping dataset containing the same 555,000 SNPs as the cases. Raw genotype data for 258 of the ALS cases and 259 of the control samples have previously been made publicly available [9], [23].

All samples were genotyped at the Laboratory of Neurogenetics, National Institute on Aging, Bethesda, MD according to the manufacturer's protocol. The resulting genotypes were visualized using the BeadStudio software package version 3.1.4 (Illumina, Inc.) using Human Genome Build 17 as reference. Any sample with a call rate below 95% were repeated on a fresh DNA aliquot and if the call rate persisted below this level the sample was excluded from the analysis.

### Copy number variant analysis

Two metrics generated during SNP chip genotyping (namely, logR ratio which is a normalized value representing the total amount of DNA hybridized to a SNP probe, and B allele frequency which represents the fraction of intensity due to the B allele) were manually inspected to determine the number of allele copies at each SNP. Using this data, the *TARDBP* locus (plus the flanking 100kb) was evaluated for structural genomic variants. A two-tailed Fisher exact test (1 degree of freedom) was then used to evaluate the significance of differences between cases and controls at each SNP. Duplications and deletions were analyzed separately. The Database of Genomic Variants (http://projects.tcag.ca/variation/, accessed 28^th^ January 2008) was reviewed to determine if identified CNV had been previously described.

### Genetic and haplotype association analysis

42 SNPs within the *TARDBP* locus and the flanking 100kb were genotyped by the Illumina HumanHap550 BeadChip and were available for analysis. SNP and haplotype association were computed for each SNP using the PLINK toolset [24]. Each SNP was required to have a call rate greater than or equal to 95% and each SNP was tested for departures from Hardy-Weinberg equilibrium. Association tests were computed using an additive model (Cochran-Armitage trend test, 1 degree of freedom), and using the three-marker sliding-window haplotype-association algorithm contained within PLINK. Odds ratios with upper and lower bound 95% confidence intervals were computed for the minor allele of each SNP.

## Results

Table 1 details the sequence variants found in the 279 ALS cases, the 806 North American controls and in the 173 African samples that were screened as part of this study. Pathogenic mutations were not identified in any of the 279 ALS cases. Five of the ALS samples (ND09546, ND10023, ND09582, ND10379, ND10379) carried the synonymous variation p.A66A (c.198 T>C) in exon 2 of *TARDBP*, but this sequence variant was likely to be a benign polymorphism as it was found in eight of the Caucasian controls and in a single Bedouin sample of the HGDP. Furthermore, the previously reported pathogenic sequence variants were not present in any of the case or control samples, though a synonymous variant involving codon 315 (p.A315A, c.945G>A) was identified in a single Caucasian control individual (ND05681).

**Table 1:** Sequence variations found in the *TARDBP* gene in the 279 ALS cases, 806 North American controls and 173 African samples.

Exon	Variant	Number of ALS cases	Number of controls	Origin of samples
2	p. A66A	5	8	1 African (Bedouin) 12 Caucasian
2	p. D65E	0	1	African (Yoruba)
3	p. A90V	0	2	Caucasian
5	p. P225P	0	4	African (1 Biaka Pygmies; 2 Mandenka; 1 Yoruba)
6	p. N352N	0	5	African (4 San; 1 Bantu)

Nineteen control samples were excluded due to poor quality genotyping on the Illumina HumanHap SNP chips (i.e. call rates <95%). Thus the final cohort for which genotyping chip data was available consisted of 279 ALS cases and 787 control individuals. Copy number analysis of the *TARDBP* locus using data generated with Illumina Infinium II HumanHap550 BeadChips did not reveal structural abnormalities affecting the TARDBP gene (±75kb) in any of the 279 cases.

Statistical analysis of association was done for 42 tagging SNPs within the *TARDBP* gene and the flanking 100kb, irrespective of Hardy-Weinberg disequilibrium or minor allele frequency. One SNP (rs12059717 located 17.kb from the gene) was excluded due to low call rate. None of the remaining 41 SNPs were significantly associated with altered risk of developing ALS under the additive model. Similarly, no haplotype significantly altering disease risk was identified (Supplementary Tables S1 and S2).

## Discussion

In this study we evaluated the role of *TARDBP* in the pathogenesis of sporadic ALS by undertaking sequence mutational analysis, as well as evaluating genomic structural variation, genetic association and haplotype association in a cohort of 279 North American ALS patients and 806 neurologically normal controls. We did not find any genetic or genomic evidence that *TARDBP* was associated with sporadic disease. Although the size of our cohort is not large enough to exclude this gene as the underlying etiology of occasional rare cases of sporadic ALS, our data indicates that mutations in *TARDBP* are not a common cause of sporadic motor neuron degeneration in the North American population (i.e. no greater than 0.4% of all cases). Although mutations in the *TARDBP* gene are rare, the protein may still play an important role in ALS. In fact, several studies have linked ALS pathogenesis to perturbations of TDP-43 nuclear trafficking, solubility and intracellular accumulation [25]–[27].

Our data agree with previous reports of a negative association between *TARDP* and ALS in 237 sporadic patients [28]. Furthermore, we did not find any case or control samples in our series that carried any of the previously reported pathogenic mutations (A315T, A382T or M337V) that have been reported in familial cases, or any of the 8 variants (D169G, G287S, G294A, Q331K, G348C, R361S, N390D and N390S) that each have been reported in single sporadic cases. Although the published segregation data strongly supports the pathogenicity of the M337V and A315T mutations within these families, the lack of further examples of the eight mutations identified within sporadic ALS cases suggests that the true pathogenic nature of these variants remains to be established. Interestingly, all the variants found and considered by the authors as pathogenic, with the exception of D169G in exon 4, are situated in exon 6; all non-synonymous but benign variants identified by us, lie outside of this exon. These mutations may alter the normal function/transport of TDP-43 or they may cause a toxic gain of function of the protein by altering its C-terminal [19].

Structural variants of the *TARDBP* gene were not a cause of sporadic ALS in our dataset. Although the density of SNPs on the Illumina HumanHap550 is not sufficient to exclude small deletions or insertions (42 SNPs over 212.9Kb, 1 SNP per 5Kb), our sequence analysis did excluded the presence of small deletions/insertions within the coding sequence and flanking introns of *TARDBP*. Finally, neither genetic association nor haplotype association with disease was observed across the *TARDBP* locus. Although this analysis was based on a relatively small number of markers, these 42 SNPs were tagging SNPs that are representative of neighboring sequence variation and extract a larger amount of genetic information than a similar number of randomly chosen SNPs.

We sequenced *TARDBP* in a cohort of 173 African samples to determine the variability of the gene in a non-Caucasian population and because this population carried the highest likelihood of finding changes. As expected, given the greater genetic variation known to exist within African populations, SNPs were more commonly present in this cohort compared to the Caucasian controls (6,9% compared to 1,1%). This observation is consistent with the older age of these populations with the consequent accumulation of sequence variants over a greater number of generations [29], [30]. Interestingly, we observed only two non-synonymous SNPs within *TARDBP* in both the African and Caucasian controls (i.e. D65E and A90V). We speculate that the biological importance of this gene has resulted in negative selective pressure against variants that alter TDP-43 amino-acid sequence.

In conclusion, our comprehensive genetic and genomic assessment of *TARDBP* failed to identify disease associated variants in the North American patients studied here. Taken together with previous reports, we conclude that mutations in *TARDBP* are not a common cause of sporadic motor neuron degeneration.

## Supporting Information

Table S1 Statistical analysis of association of the 41 tagging SNPs within the TARDBP gene and flanking 100kb and the risk of disease. None of the 41 tagging SNPs was significantly associated with an altered risk of developing ALS. CHR: Chromosome; bp position: base pairs position relative to human genome build 36; p<0.05 values were considered statistically significant.
(0.10 MB DOC)

Table S2 Statistical analysis of association of haplotypes within the TARDBP locus and the risk of disease. None of the haplotypes was significantly associated with an altered risk of developing ALS. p<0.05 values were considered statistically significant.
(0.24 MB DOC)
