# Supplementary material for: TDP-43 Is Not a Common Cause of Sporadic Amyotrophic Lateral Sclerosis
Source: PLoS One. 2008 Jun 11;3(6):e2450. doi: 10.1371/journal.pone.0002450 (PMC2408729; doi:10.1371/journal.pone.0002450)
Supplement: Table S1 — Statistical analysis of association of the 41 tagging SNPs within the TARDBP gene and flanking 100kb and the risk of disease. None of the 41 tagging SNPs was significantly associated with an altered risk of developing ALS. CHR: Chromosome; bp position: base pairs position relative to human genome build 36; p<0.05 values were considered statistically significant. (0.10 MB DOC) [file pone.0002450.s001.doc]

**Supplementary Table S1**

Statistical analysis of association of the 41 tagging SNPs within the *TARDBP* gene and flanking 100kb and the risk of disease

| CHR | bp position  (Build 36) | SNP | Affected | Unaffected | *P* value (1df) |
| --- | --- | --- | --- | --- | --- |
| 1 | 10902996 | rs10864479 | 224/318 | 666/920 | 0.7867 |
| 1 | 10908595 | rs10157927 | 114/428 | 322/1266 | 0.7051 |
| 1 | 10910961 | rs1280975 | 66/476 | 180/1408 | 0.6026 |
| 1 | 10912328 | rs1280972 | 155/387 | 471/1117 | 0.6423 |
| 1 | 10913076 | rs1280970 | 267/275 | 783/805 | 0.9857 |
| 1 | 10914810 | rs4845964 | 214/328 | 622/966 | 0.8983 |
| 1 | 10915264 | rs2387422 | 215/327 | 633/953 | 0.9193 |
| 1 | 10918217 | rs2486671 | 138/404 | 398/1188 | 0.8654 |
| 1 | 10918828 | rs1280984 | 115/427 | 353/1233 | 0.6135 |
| 1 | 10920574 | rs12748162 | 136/406 | 362/1218 | 0.2929 |
| 1 | 10926370 | rs2387698 | 202/340 | 579/1005 | 0.7708 |
| 1 | 10927993 | rs12066915 | 151/391 | 455/1133 | 0.7207 |
| 1 | 10932775 | rs744921 | 36/506 | 137/1451 | 0.1465 |
| 1 | 10933866 | rs1925666 | 93/449 | 275/1313 | 0.9326 |
| 1 | 10934542 | rs12755921 | 238/304 | 733/855 | 0.3411 |
| 1 | 10936754 | rs6656310 | 154/384 | 381/1189 | 0.04351 |
| 1 | 10945119 | rs1281009 | 19/523 | 49/1539 | 0.6407 |
| 1 | 10945268 | rs1281008 | 88/454 | 289/1299 | 0.2975 |
| 1 | 10950644 | rs6540959 | 122/420 | 390/1198 | 0.3354 |
| 1 | 10958619 | rs6659231 | 137/405 | 387/1201 | 0.6703 |
| 1 | 10958661 | rs11121663 | 151/391 | 429/1159 | 0.6995 |
| 1 | 10958903 | rs11121664 | 145/397 | 412/1176 | 0.7111 |
| 1 | 10967093 | rs2003046 | 114/426 | 347/1241 | 0.7153 |
| 1 | 10972002 | rs11576658 | 115/427 | 344/1244 | 0.8272 |
| 1 | 10981121 | rs9430161 | 111/431 | 308/1280 | 0.5775 |
| 1 | 10987367 | rs6704113 | 14/528 | 45/1543 | 0.7595 |
| 1 | 10994341 | rs11121675 | 162/378 | 535/1053 | 0.1151 |
| 1 | 10997309 | rs11121676 | 81/461 | 260/1326 | 0.4268 |
| 1 | 11004918 | rs12744501 | 169/373 | 564/1022 | 0.06601 |
| 1 | 11008686 | rs3765896 | 82/460 | 265/1323 | 0.4006 |
| 1 | 11013343 | rs2273348 | 88/452 | 302/1286 | 0.1609 |
| 1 | 11020983 | rs1033638 | 83/459 | 264/1302 | 0.4056 |
| 1 | 11025182 | rs12711521 | 88/454 | 303/1281 | 0.1372 |
| 1 | 11041705 | rs7548659 | 111/431 | 343/1237 | 0.5542 |
| 1 | 11045895 | rs12121344 | 8/534 | 38/1550 | 0.2098 |
| 1 | 11066483 | rs2802211 | 140/402 | 421/1167 | 0.7571 |
| 1 | 11075368 | rs2273337 | 9/533 | 37/1549 | 0.3581 |
| 1 | 11100568 | rs6540964 | 141/401 | 419/1169 | 0.8664 |
| 1 | 11100979 | rs2536 | 11/531 | 39/1549 | 0.582 |
| 1 | 11103942 | rs2275525 | 10/532 | 39/1549 | 0.4169 |
| 1 | 11105492 | rs17036350 | 10/532 | 39/1549 | 0.4169 |

None of the 41 tagging SNPs was significantly associated with an altered risk of developing ALS. CHR: Chromosome; bp position: base pairs position relative to human genome build 36; p<0.05 values were considered statistically significant.
