# Supplementary material for: TDP-43 Is Not a Common Cause of Sporadic Amyotrophic Lateral Sclerosis
Source: PLoS One. 2008 Jun 11;3(6):e2450. doi: 10.1371/journal.pone.0002450 (PMC2408729; doi:10.1371/journal.pone.0002450)
Supplement: Table S2 — Statistical analysis of association of haplotypes within the TARDBP locus and the risk of disease. None of the haplotypes was significantly associated with an altered risk of developing ALS. p<0.05 values were considered statistically significant. (0.24 MB DOC) [file pone.0002450.s002.doc]

**Supplementary Table S2**

Statistical analysis of association of haplotypes within the *TARDBP* locus and the risk of disease

| **SNPS** | **Haplotype** | **Frequency  affected** | **Frequency  unaffected** | ***P* value (1df)** |
| --- | --- | --- | --- | --- |
| rs2387698|rs12066915|rs744921 | AAC | 0.08978 | 0.08756 | 0.8752 |
| rs6540959|rs6659231|rs11121663 | AAC | 0.02773 | 0.02717 | 0.9456 |
| rs1280984|rs12748162|rs2387698 | AAG | 0.2531 | 0.2291 | 0.2583 |
| rs1925666|rs12755921|rs6656310 | AAT | 0.4366 | 0.4586 | 0.3753 |
| rs6540959|rs6659231|rs11121663 | AAT | 0.688 | 0.6902 | 0.9213 |
| rs1281008|rs6540959|rs6659231 | ACA | 0.01881 | 0.0272 | 0.2821 |
| rs12066915|rs744921|rs1925666 | ACA | 0.1487 | 0.154 | 0.7685 |
| rs1280984|rs12748162|rs2387698 | ACA | 0.3659 | 0.3636 | 0.9239 |
| rs3765896|rs2273348|rs1033638 | ACC | 0.8391 | 0.8117 | 0.1539 |
| rs2387698|rs12066915|rs744921 | ACC | 0.2582 | 0.2513 | 0.7506 |
| rs1281008|rs6540959|rs6659231 | ACG | 0.1389 | 0.1527 | 0.436 |
| rs1280984|rs12748162|rs2387698 | ACG | 0.1787 | 0.189 | 0.5941 |
| rs12066915|rs744921|rs1925666 | ACG | 0.1281 | 0.1289 | 0.9627 |
| rs6659231|rs11121663|rs11121664 | ACT | 0.02773 | 0.02732 | 0.9599 |
| rs2387698|rs12066915|rs744921 | ACT | 0.02407 | 0.02419 | 0.9873 |
| rs12748162|rs2387698|rs12066915 | AGA | 0.05915 | 0.05383 | 0.6394 |
| rs6540959|rs6659231|rs11121663 | AGC | 0.06065 | 0.03839 | 0.02938 |
| rs1925666|rs12755921|rs6656310 | AGC | 0.2834 | 0.2398 | 0.04384 |
| rs12711521|rs7548659|rs12121344 | AGC | 0.04259 | 0.02918 | 0.1297 |
| rs6704113|rs11121675|rs11121676 | AGC | 0.1543 | 0.1723 | 0.3332 |
| rs12748162|rs2387698|rs12066915 | AGC | 0.1918 | 0.1757 | 0.4002 |
| rs1925666|rs12755921|rs6656310 | AGT | 0.1076 | 0.1275 | 0.2239 |
| rs6704113|rs11121675|rs11121676 | AGT | 0.148 | 0.1635 | 0.3957 |
| rs3765896|rs2273348|rs1033638 | ATC | 0.009332 | 0.02149 | 0.06926 |
| rs6704113|rs11121675|rs11121676 | ATC | 0.6729 | 0.6376 | 0.1391 |
| rs12755921|rs6656310|rs1281009 | ATC | 0.4379 | 0.4622 | 0.3289 |
| rs12711521|rs7548659|rs12121344 | ATC | 0.7963 | 0.782 | 0.4832 |
| rs6659231|rs11121663|rs11121664 | ATC | 0.01479 | 0.01596 | 0.8501 |
| rs4845964|rs2387422|rs2486671 | ATC | 0.3948 | 0.3905 | 0.8596 |
| rs2003046|rs11576658|rs9430161 | ATG | 0.02882 | 0.0381 | 0.3175 |
| rs6659231|rs11121663|rs11121664 | ATT | 0.7061 | 0.7143 | 0.7146 |
| rs2802211|rs2273337|rs6540964 | ATT | 0.7412 | 0.7373 | 0.8583 |
| rs2003046|rs11576658|rs9430161 | ATT | 0.1798 | 0.1771 | 0.8913 |
| rs744921|rs1925666|rs12755921 | CAA | 0.4391 | 0.4616 | 0.3644 |
| rs2486671|rs1280984|rs12748162 | CAA | 0.2129 | 0.2047 | 0.6881 |
| rs12748162|rs2387698|rs12066915 | CAA | 0.09101 | 0.08999 | 0.9429 |
| rs1281009|rs1281008|rs6540959 | CAC | 0.1243 | 0.1496 | 0.1472 |
| rs12744501|rs3765896|rs2273348 | CAC | 0.1617 | 0.1752 | 0.4742 |
| rs2486671|rs1280984|rs12748162 | CAC | 0.5385 | 0.5522 | 0.583 |
| rs12748162|rs2387698|rs12066915 | CAC | 0.2817 | 0.2752 | 0.7717 |
| rs744921|rs1925666|rs12755921 | CAG | 0.3229 | 0.279 | 0.05178 |
| rs1033638|rs12711521|rs7548659 | CAG | 0.04436 | 0.02982 | 0.1047 |
| rs12744501|rs3765896|rs2273348 | CAT | 0.01119 | 0.0235 | 0.08134 |
| rs2536|rs2275525|rs17036350 | CAT | 0.01848 | 0.02456 | 0.4158 |
| rs6540959|rs6659231|rs11121663 | CAT | 0.03292 | 0.0397 | 0.4766 |
| rs1033638|rs12711521|rs7548659 | CAT | 0.7948 | 0.7813 | 0.5094 |
| rs12121344|rs2802211|rs2273337 | CAT | 0.7431 | 0.7349 | 0.7089 |
| rs1280970|rs4845964|rs2387422 | CAT | 0.01226 | 0.01069 | 0.7625 |
| rs11121664|rs2003046|rs11576658 | CAT | 0.05297 | 0.05345 | 0.9657 |
| rs2273348|rs1033638|rs12711521 | CCA | 0.8376 | 0.8096 | 0.145 |
| rs6540964|rs2536|rs2275525 | CCA | 0.01655 | 0.02439 | 0.2875 |
| rs12066915|rs744921|rs1925666 | CCA | 0.6152 | 0.5904 | 0.3103 |
| rs2387422|rs2486671|rs1280984 | CCA | 0.1388 | 0.1453 | 0.7071 |
| rs11121676|rs12744501|rs3765896 | CCA | 0.03591 | 0.03765 | 0.8542 |
| rs11121663|rs11121664|rs2003046 | CCA | 0.04504 | 0.04383 | 0.9067 |
| rs2273337|rs6540964|rs2536 | CCC | 0.01667 | 0.02393 | 0.3211 |
| rs1280975|rs1280972|rs1280970 | CCC | 0.286 | 0.2962 | 0.6532 |
| rs11121663|rs11121664|rs2003046 | CCC | 0.2117 | 0.2046 | 0.7252 |
| rs11121664|rs2003046|rs11576658 | CCC | 0.2117 | 0.2064 | 0.7938 |
| rs1033638|rs12711521|rs7548659 | CCG | 0.009242 | 0.02272 | 0.04908 |
| rs6656310|rs1281009|rs1281008 | CCG | 0.2509 | 0.2111 | 0.05544 |
| rs11121676|rs12744501|rs3765896 | CCG | 0.1399 | 0.1601 | 0.2674 |
| rs1280972|rs1280970|rs4845964 | CCG | 0.2809 | 0.2923 | 0.6171 |
| rs12066915|rs744921|rs1925666 | CCG | 0.04392 | 0.04519 | 0.902 |
| rs2003046|rs11576658|rs9430161 | CCG | 0.7691 | 0.7694 | 0.9894 |
| rs2003046|rs11576658|rs9430161 | CCT | 0.02232 | 0.01538 | 0.2846 |
| rs1281009|rs1281008|rs6540959 | CGA | 0.7737 | 0.7538 | 0.3517 |
| rs12748162|rs2387698|rs12066915 | CGA | 0.1284 | 0.1427 | 0.4071 |
| rs11576658|rs9430161|rs6704113 | CGA | 0.7454 | 0.7459 | 0.9825 |
| rs12711521|rs7548659|rs12121344 | CGC | 0.1481 | 0.1655 | 0.3439 |
| rs6540959|rs6659231|rs11121663 | CGC | 0.1907 | 0.2045 | 0.4903 |
| rs12748162|rs2387698|rs12066915 | CGC | 0.248 | 0.2626 | 0.5028 |
| rs1280970|rs4845964|rs2387422 | CGC | 0.2802 | 0.2893 | 0.6832 |
| rs1281009|rs1281008|rs6540959 | CGC | 0.06865 | 0.06629 | 0.8499 |
| rs11576658|rs9430161|rs6704113 | CGG | 0.02272 | 0.02465 | 0.8009 |
| rs744921|rs1925666|rs12755921 | CGG | 0.1716 | 0.1732 | 0.9327 |
| rs12711521|rs7548659|rs12121344 | CGT | 0.01296 | 0.02337 | 0.1425 |
| rs12744501|rs3765896|rs2273348 | CGT | 0.1422 | 0.1597 | 0.3333 |
| rs1280970|rs4845964|rs2387422 | CGT | 0.2002 | 0.193 | 0.7164 |
| rs12121344|rs2802211|rs2273337 | CGT | 0.2421 | 0.2412 | 0.964 |
| rs11121676|rs12744501|rs3765896 | CTA | 0.6819 | 0.6402 | 0.08154 |
| rs12066915|rs744921|rs1925666 | CTA | 0.06408 | 0.08155 | 0.1885 |
| rs2387422|rs2486671|rs1280984 | CTA | 0.04645 | 0.03514 | 0.2359 |
| rs11576658|rs9430161|rs6704113 | CTA | 0.02223 | 0.01584 | 0.3272 |
| rs6656310|rs1281009|rs1281008 | CTA | 0.03231 | 0.0257 | 0.4181 |
| rs1280975|rs1280972|rs1280970 | CTC | 0.2066 | 0.1972 | 0.6359 |
| rs11121663|rs11121664|rs2003046 | CTC | 0.02184 | 0.02233 | 0.9477 |
| rs2387422|rs2486671|rs1280984 | CTG | 0.2091 | 0.2157 | 0.7485 |
| rs6540964|rs2536|rs2275525 | CTG | 0.2446 | 0.2395 | 0.8114 |
| rs1280975|rs1280972|rs1280970 | CTT | 0.3856 | 0.3932 | 0.7547 |
| rs1281008|rs6540959|rs6659231 | GAA | 0.7127 | 0.7154 | 0.9034 |
| rs2387698|rs12066915|rs744921 | GAC | 0.1881 | 0.1962 | 0.6788 |
| rs1281008|rs6540959|rs6659231 | GAG | 0.06098 | 0.03838 | 0.02732 |
| rs9430161|rs6704113|rs11121675 | GAG | 0.2076 | 0.2394 | 0.1293 |
| rs9430161|rs6704113|rs11121675 | GAT | 0.5639 | 0.5437 | 0.4145 |
| rs1281008|rs6540959|rs6659231 | GCA | 0.01434 | 0.01308 | 0.8259 |
| rs12755921|rs6656310|rs1281009 | GCC | 0.2534 | 0.218 | 0.09107 |
| rs11121675|rs11121676|rs12744501 | GCC | 0.147 | 0.1704 | 0.208 |
| rs2802211|rs2273337|rs6540964 | GCC | 0.01664 | 0.02321 | 0.3635 |
| rs2387698|rs12066915|rs744921 | GCC | 0.3985 | 0.3822 | 0.4994 |
| rs10157927|rs1280975|rs1280972 | GCC | 0.2794 | 0.2914 | 0.5959 |
| rs4845964|rs2387422|rs2486671 | GCC | 0.1421 | 0.1511 | 0.6119 |
| rs6659231|rs11121663|rs11121664 | GCC | 0.2514 | 0.2424 | 0.674 |
| rs1280984|rs12748162|rs2387698 | GCG | 0.2024 | 0.2183 | 0.4386 |
| rs7548659|rs12121344|rs2802211 | GCG | 0.187 | 0.1936 | 0.7398 |
| rs1281008|rs6540959|rs6659231 | GCG | 0.0543 | 0.05321 | 0.9222 |
| rs2387698|rs12066915|rs744921 | GCT | 0.04137 | 0.05857 | 0.1271 |
| rs12755921|rs6656310|rs1281009 | GCT | 0.03149 | 0.0238 | 0.3312 |
| rs4845964|rs2387422|rs2486671 | GCT | 0.2546 | 0.2492 | 0.8022 |
| rs10157927|rs1280975|rs1280972 | GCT | 0.3947 | 0.3976 | 0.9062 |
| rs10864479|rs10157927|rs1280975 | GGC | 0.3694 | 0.3769 | 0.7578 |
| rs1925666|rs12755921|rs6656310 | GGT | 0.1724 | 0.174 | 0.9287 |
| rs9430161|rs6704113|rs11121675 | GGT | 0.02423 | 0.02468 | 0.9528 |
| rs11121675|rs11121676|rs12744501 | GTC | 0.143 | 0.1609 | 0.3282 |
| rs12755921|rs6656310|rs1281009 | GTC | 0.2772 | 0.2959 | 0.4085 |
| rs10864479|rs10157927|rs1280975 | GTC | 0.2123 | 0.2018 | 0.603 |
| rs6704113|rs11121675|rs11121676 | GTC | 0.02479 | 0.02651 | 0.8287 |
| rs2802211|rs2273337|rs6540964 | GTC | 0.2421 | 0.2395 | 0.9005 |
| rs4845964|rs2387422|rs2486671 | GTC | 0.2085 | 0.2092 | 0.9718 |
| rs7548659|rs12121344|rs2802211 | GTG | 0.01481 | 0.02394 | 0.2077 |
| rs3765896|rs2273348|rs1033638 | GTT | 0.1516 | 0.1668 | 0.4085 |
| rs10157927|rs1280975|rs1280972 | GTT | 0.1229 | 0.1142 | 0.5869 |
| rs2486671|rs1280984|rs12748162 | TAA | 0.03893 | 0.02574 | 0.1172 |
| rs12744501|rs3765896|rs2273348 | TAC | 0.6849 | 0.6415 | 0.06876 |
| rs1281009|rs1281008|rs6540959 | TAC | 0.0334 | 0.0303 | 0.7209 |
| rs744921|rs1925666|rs12755921 | TAG | 0.06642 | 0.08627 | 0.1441 |
| rs9430161|rs6704113|rs11121675 | TAG | 0.09181 | 0.0964 | 0.7538 |
| rs9430161|rs6704113|rs11121675 | TAT | 0.1125 | 0.09583 | 0.2647 |
| rs11121664|rs2003046|rs11576658 | TAT | 0.1556 | 0.1618 | 0.7357 |
| rs1280970|rs4845964|rs2387422 | TAT | 0.3826 | 0.381 | 0.9481 |
| rs6656310|rs1281009|rs1281008 | TCA | 0.1236 | 0.1442 | 0.2321 |
| rs11121676|rs12744501|rs3765896 | TCA | 0.1423 | 0.1621 | 0.2786 |
| rs7548659|rs12121344|rs2802211 | TCA | 0.7407 | 0.7347 | 0.7839 |
| rs2387422|rs2486671|rs1280984 | TCA | 0.6056 | 0.6038 | 0.9404 |
| rs2273348|rs1033638|rs12711521 | TCC | 0.009225 | 0.02233 | 0.05366 |
| rs11121675|rs11121676|rs12744501 | TCC | 0.02629 | 0.02715 | 0.9154 |
| rs11121664|rs2003046|rs11576658 | TCC | 0.5797 | 0.5784 | 0.9557 |
| rs6656310|rs1281009|rs1281008 | TCG | 0.5932 | 0.6189 | 0.2915 |
| rs7548659|rs12121344|rs2802211 | TCG | 0.05741 | 0.04778 | 0.3764 |
| rs1033638|rs12711521|rs7548659 | TCG | 0.1516 | 0.1661 | 0.4277 |
| rs1280972|rs1280970|rs4845964 | TCG | 0.2053 | 0.1955 | 0.6236 |
| rs11121675|rs11121676|rs12744501 | TCT | 0.6837 | 0.6416 | 0.07812 |
| rs10157927|rs1280975|rs1280972 | TCT | 0.203 | 0.1968 | 0.7565 |
| rs2273337|rs6540964|rs2536 | TCT | 0.2444 | 0.2401 | 0.8367 |
| rs11576658|rs9430161|rs6704113 | TGA | 0.02729 | 0.03683 | 0.2935 |
| rs12121344|rs2802211|rs2273337 | TGC | 0.01479 | 0.02393 | 0.2066 |
| rs2536|rs2275525|rs17036350 | TGC | 0.9815 | 0.9754 | 0.4158 |
| rs1280970|rs4845964|rs2387422 | TGC | 0.1165 | 0.1102 | 0.6871 |
| rs2486671|rs1280984|rs12748162 | TGC | 0.2097 | 0.2173 | 0.7102 |
| rs10864479|rs10157927|rs1280975 | TGC | 0.3088 | 0.3145 | 0.8065 |
| rs1280970|rs4845964|rs2387422 | TGT | 0.008283 | 0.01572 | 0.2007 |
| rs10864479|rs10157927|rs1280975 | TGT | 0.1095 | 0.1068 | 0.8638 |
| rs11121663|rs11121664|rs2003046 | TTA | 0.1578 | 0.1641 | 0.7335 |
| rs11576658|rs9430161|rs6704113 | TTA | 0.1824 | 0.1768 | 0.771 |
| rs1280972|rs1280970|rs4845964 | TTA | 0.3873 | 0.3846 | 0.914 |
| rs2273348|rs1033638|rs12711521 | TTC | 0.1531 | 0.1681 | 0.4174 |
| rs11121663|rs11121664|rs2003046 | TTC | 0.5636 | 0.5652 | 0.95 |
| rs6540964|rs2536|rs2275525 | TTG | 0.7389 | 0.7361 | 0.9 |
| rs1280972|rs1280970|rs4845964 | TTG | 0.1265 | 0.1276 | 0.9488 |
| rs1280975|rs1280972|rs1280970 | TTT | 0.1218 | 0.1134 | 0.5996 |
| rs2273337|rs6540964|rs2536 | TTT | 0.7389 | 0.736 | 0.8959 |

None of the haplotypes was significantly associated with an altered risk of developing ALS. p<0.05 values were considered statistically significant.
